# Plant-based model for the visual evaluation of electroporated area after irreversible electroporation and its comparison to in-vivo animal data

**DOI:** 10.1177/00368504231156294

**Published:** 2023-02-20

**Authors:** Kim. H. K. Lindelauf, Athul Thomas, Marco Baragona, Ali Jouni, Teresa Nolte, Federico Pedersoli, Joachim Pfeffer, Martin Baumann, Ralph. T. H. Maessen, Andreas Ritter

**Affiliations:** 1 Department of Diagnostic and Interventional Radiology, University Hospital 39058 RWTH Aachen, Aachen, Germany; 2 60994 Philips Research, Eindhoven, The Netherlands; 3 Institute of Applied Medical Engineering, 9165 RWTH Aachen University, Aachen, Germany

**Keywords:** electroporation, irreversible electroporation, IRE, plant-based model, animal experiment, electroporated area

## Abstract

Electroporation (EP) is widely used in medicine, such as cancer treatment, in form of electrochemotherapy or irreversible electroporation (IRE). For EP device testing, living cells or tissue inside a living organism (including animals) are needed. Plant-based models seem to be a promising alternative to substitute animal models in research. The aim of this study is to find a suitable plant-based model for visual evaluation of IRE, and to compare the geometry of electroporated areas with in-vivo animal data.

For this purpose, a variety of fruit and vegetables were selected and visually evaluated after 0/1/2/4/6/8/12/16/24 h post-EP. Apple and potato were found to be suitable models as they enabled a visual evaluation of the electroporated area. For these models, the size of the electroporated area was determined after 0/1/2/4/6/8/12/16/24 h. For apples, a well-defined electroporated area was visual within two hours, while in potatoes it reached a plateau after eight hours only. The electroporated area of apple, which showed the fastest visual results was then compared to a retrospectively evaluated swine liver IRE dataset which had been obtained for similar conditions. The electroporated area of the apple and swine liver both showed a spherical geometry of comparable size. For all experiments, the standard protocol for human liver IRE was followed. To conclude, potato and apple were found to be suitable plant-based models for the visual evaluation of electroporated area after irreversible EP, with apple being the best choice for fast visual results. Given the comparable range, the size of the electroporated area of the apple may be promising as a quantitative predictor in animal tissue. Even if plant-based models cannot completely replace animal experiments, they can be used in the early stages of EP device development and testing, decreasing animal experiments to the necessary minimum.

## Introduction

Electroporation (EP) is the biological effect of creating nanopores in the cell membrane of biological tissues by the application of a pulsating electric field.^1,2^ The effect of pulsating electric fields is similar on plant and animal cells due to the fact that cell walls in plant tissue do not prevent the electric field from destabilizing the cell membrane and cause EP.^3^ These pores, which increase the cellular permeability, can either be self-repairing (reversible EP, RE) or not, which leads to cell death (irreversible EP, IRE).^4^ Several electrical pulse parameters determine reversible or irreversible EP (i.e. electric field strength, number of pulses, amplitude, pulse length).^5^ The spatial extent of EP-induced damage during IRE will be referred to as the electroporated area.

EP is increasingly used in medicine, such as during interventional procedures for minimal-invasive cancer treatment, but also in biological methods like CRISPR/Cas9.^6^ To test thoroughly EP devices and relate the settings to the corresponding outcome, EP experiments need to be carried out on living cells or tissue inside living organisms. This is different from thermal ablation procedures which allow for easier testing in ex-vivo tissues.^7^ Hence, IRE device development often requires animal trials.^8^ However, as per the principles of the 3Rs (replacement, reduction, and refinement), established for more ethical use of animals in product testing and scientific research, there is a continuing need for alternative models in order to harm fewer animals while obtaining similar scientific outcomes.^9^ Numerical models can be considered as possible replacement models.^10–12^ Plant-based models have also gained much interest over the past years and seem to be a promising alternative to animal testing in research.

Every biological cell of animals and plants is different in its structure, size, and shape since it has different roles in nature. Many plant cells have cellulose walls and chloroplasts, whereas, in animals, the plasma membrane replaces these cell walls.^13^ However, despite the differences in cell structure, while considering EP from the electrical point of view, both animal cells and plant cells are very similar: a biological cell can be seen as a sphere that is electrically isolated by its outer covering named a plasma membrane, which creates an electrical potential difference between the inside and outside of the cell. This electrical potential difference causes the transmembrane voltage. So, to a certain level of approximation, they both can be represented by the same electrical equivalent.^14^ The breakdown of the membrane in the first few microseconds after EP was found to be similar in both animal tissues and plant tissues. Angersbach et al.^15^ observed this in potato, apple, and ex-vivo fish tissues. Slight membrane damage was initiated at 150–200 V/cm and significant membrane damage occurred above 400 V/cm. Pulsed electric field parameters have a crucial role in successful non-thermal EP of the cell membrane.^16^

Potatoes have been used as a plant-based model for the visualisation of the electroporated area.^17–19^ In 2010, Hjouj et al. proposed to visualize the electroporated area in potato faster by magnetic resonance imaging at different time points post-EP, as an alternative option to visual evaluation of melanin accumulation, which usually takes 6 to 12 h to develop post-EP.^20^ More recently, in 2021, Jeong et al.^21^ analysed electroporated areas at various field strengths and time points by means of photographic images and 23,5-triphenyltetrazolium chloride TTC staining methods. The sample preparation for both melanin browning and the TTC staining methods took 48 h. TTC staining visualized the electroporated area faster, within 3 h, and with well-defined margins, whereas melanin accumulation took 12 h post-EP. Faster staining methods are also available for potatoes, like commercial food dyes.^22^ However, most studies so far focused on better visualisation of the electroporated area in potatoes using advanced visualisation methods, but do not explore alternative plant-based models that may give better and faster results visually.

Thus, this study aimed to find a suitable plant-based model for the visual evaluation of the electroporated area after irreversible EP as support for EP device development and testing. In addition, for the best model, we aimed to explore the correlation of the electroporated area to in-vivo animal data to check if the data could also be used as qualitative (geometry) and quantitative (electroporated area) predictor for IRE results in animal tissue.

## Materials and Methods

### Best plant-based model for electroporation

To determine suitable plant-based models for EP device development and testing, the following fruits and vegetables were selected for EP experiments; potato (Solanum tuberosum var. Jazzy), apple (Malus domestica var.Cripps Pink), sweet potato (Ipomoea batatas), pear (Pyrus communis), eggplant (Solanum melongena), carrot (Daucus carota), zucchini (Cucurbita pepo var. cylindrica), cucumber (Cucumis sativus), celeriac (Apium graveolens), and kohlrabi (Brassica oleracea var. gongylodes). All vegetables and fruits used for EP were obtained from the house brand of the EDEKA supermarket group, except for the apples (Pink Lady).

**Figure 1.**
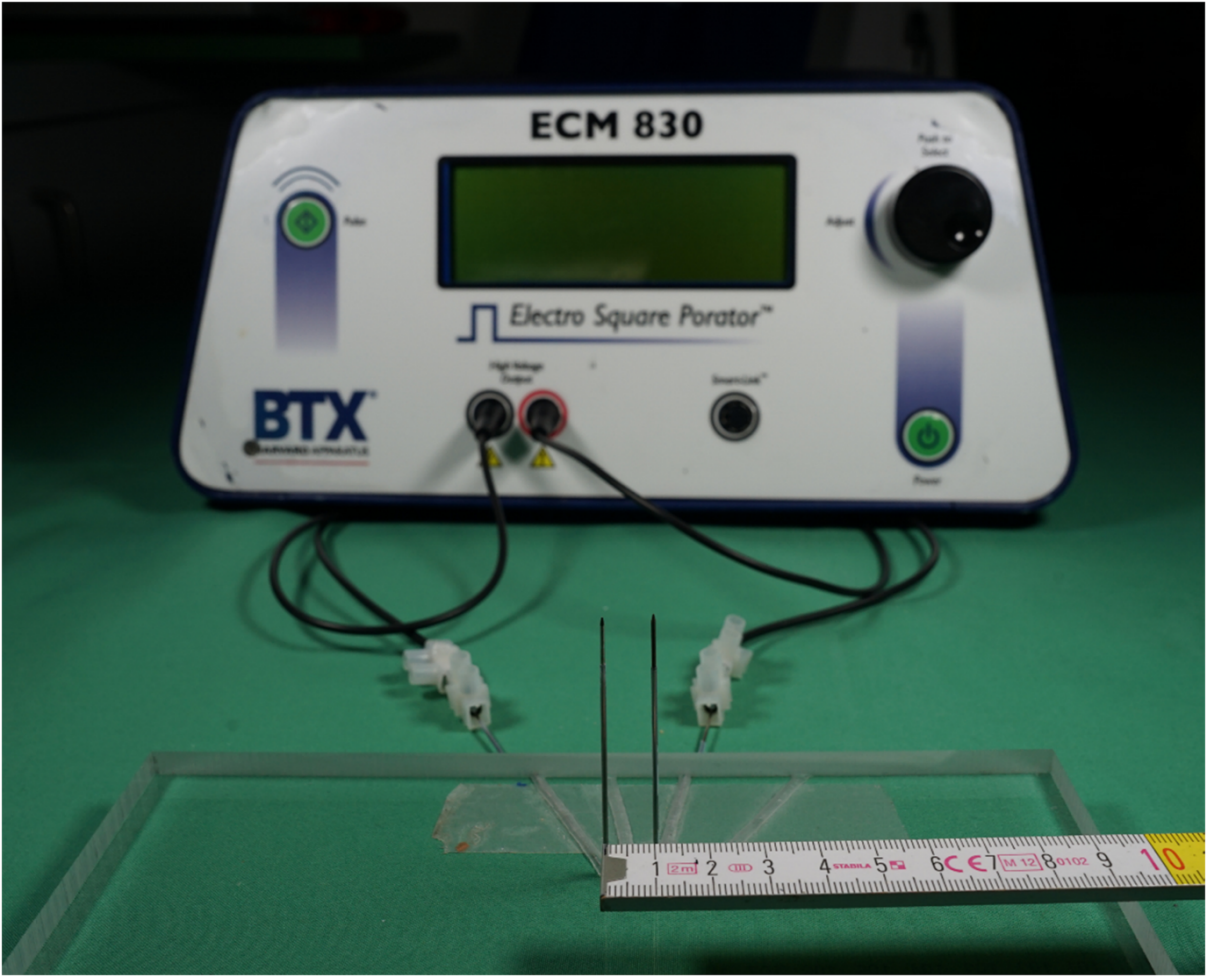
Overview of EP setup with two-point needle device used for plant-based model screening.

The samples (*N* = 5) were electroporated with a custom-made two-point needle device powered by an ECM 830 Square Wave Electroporation System (BTX, Holliston, US) (Figure 1). The distance between these stainless-steel needle electrodes, with a diameter of 1 mm, was set to 1 cm. The tip of the needles was inserted up to a depth of 1 cm into each sample. The rest of the needle was insulated with heated PTFE Teflon tubing to avoid other interactions with the surroundings. The electroporator applied 70 pulses with a voltage of 1000 V (*E*_max_ = 1 kV/cm between the electrodes), 100 *µ*s pulse length and 100 ms interval. A corresponding non-electroporated sample with the needle impression served as a control.

After EP, each sample was cut parallel to the plane defined by the two-point needle electrode for visual evaluation of the electroporated area 0, 1, 2, 4, 6, 8, 12, 16, 24 post EP. The electroporated area within the slices was photographed with a Sony ILCE-7M2 camera with exposure settings; ISO: 400, aperture: F11, shutter speed: 1/60 s. The camera was fixed on a tripod where the distance from the sample to the camera was around 40 cm. The lens used was a Sony FE SEL 24–70 mm 2.8 GM II with a constant focal length, 70 mm. The sample and a reference scale were placed between two constant 10 Watt powered light sources (NEEWER ZC-10S - LED lights), where the distance between light sources was around 40 cm at the height of 30 cm from the table.

Samples were stored at room temperature. In addition, for the best visual models, apple and potato, the size of the electroporated area in cm^2^ was determined for *N* = 5 samples and all time points. Images were analysed using the free & open-source image editor, GNU Image Manipulation Program (GIMP 2.10). The electroporated area was selected by manual segmentation using the free select tool and segmented pixels were counted. Pixel count was transformed to cm^2^ with help from the reference scale. The seed-containing core of the apple was excluded.

### Comparison of plant-based model and in-vivo animal data

To determine the correlation of the electroporated area in apple, which was chosen to be the best plant-based sample for immediate results, to previously acquired in-vivo animal data [Landesamt für Natur, Umwelt und Verbraucherschutz Nordrhein-Westfalen (LANUV); 84-02.04.2012.A155], *N* = 10 apples were electroporated and the geometry and size of the electroporated area were compared to DICOM data from swine liver. For both apples and swine liver, a two-point needle device was used, with a 1 mm needle diameter and a distance of 2 cm between needles. The needle parameters were similar in both experiments with an active length of around 1.5 cm. In addition, the following pulse parameters were used: the electroporator applied a voltage of 3000 V, 70 pulses, 100 *µ*s pulse length and 100 ms interval.

The swine liver was electroporated with the Nanoknife System (AngioDynamics, New York, US). Needles were positioned parallel with the help of a Siemens SOMATOM Definition Flash CT Scanner (Siemens, Munich, DE). One day after the EP, the electroporated area of the swine liver was observed using a Philips Achieva 1.5 T magnetic resonance imaging (MRI) scanner (Philips, Eindhoven, NL). MR images were acquired with a T1-weighted gradient echo sequence (field of view = 360 mm, voxel size (240 × 167 × 7) mm^3^, TR = 178 ms, TE = 2.3 ms, flip angle = 80°, number of averages = 1). For this retrospective study, the electroporated area was manually segmented and determined using 3D Slicer.^23^ For optimal contrast during manual segmentation, the ‘blue ocean’ colour scale was chosen for better visualisation.

The apples were electroporated with the ECM 830 Square Wave Electroporation System (BTX, Holliston, US). After EP, the apples were split open into two different planes, parallel (*N* = 5) and orthogonal (*N* = 5) to the electrodes. The electroporated area was photographed with the Sony ILCE-7M2 camera and lighting setup as described above. The 2 h post-EP images were selected for semi-automatic evaluation of the electroporated area in GIMP 2.10. First, the images were converted to 8-bit greyscale. Per image, two circular regions of interest with a radius of 100 pixels were chosen (one within the electroporated zone, one in the normal apple tissue) and histograms of the grey value distribution within these regions were obtained, showing each a distinct peak. Peak centre grey values of electroporated and normal apple tissue were determined and averaged between the ten samples. A global threshold was then fixed at a grey value of 164, corresponding to 25% of the distance between the two mean peak centre grey values, and the electroporated area in every image was measured after binary thresholding. In the case of apple, this method could be used to automatize segmentations because the resulting segmented area matched well with the visual electroporated area. The results in apple were compared to those in swine liver.

## Results

### Best plant-based model for electroporation

**Figure 2.**
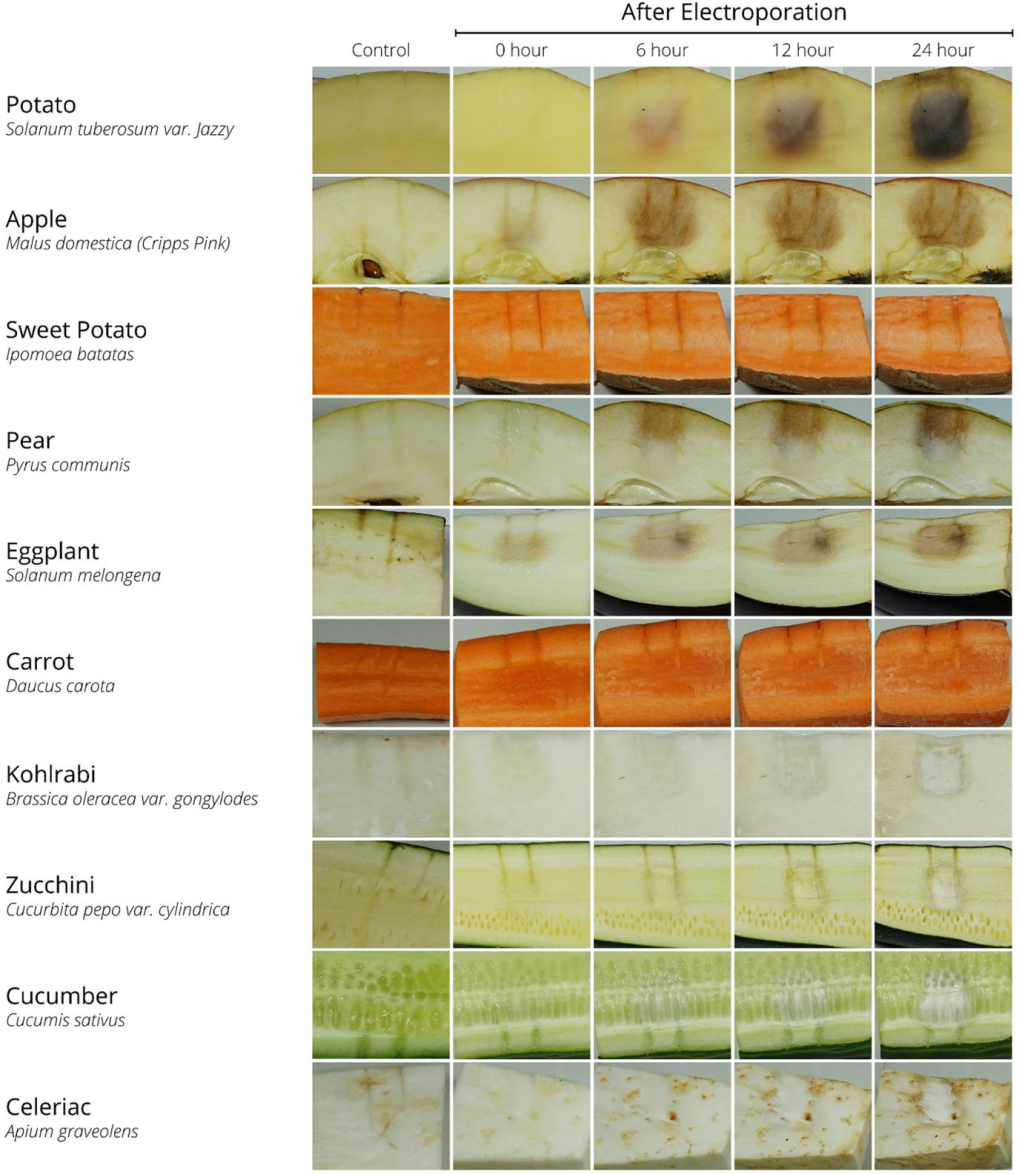
Visual representation of plant-based samples at 0, 6, 12 and 24 h post EP. EP pulse parameters included 1000 V, 70 pulses, 100 *µ*s pulse length, and 100 ms interval with a 1 cm distance between the two-needle electrodes. Non-electroporated samples with needle impression (0 h) served as control. *N* = 5.

After exposure to an electrical potential of 1000 V, 70 pulses, 100 *µ*s pulse length, and 100 ms interval, the following visual changes could be observed within the plant-based samples (Figure 2).

Directly after EP, there is a subtle brown discoloration of the electroporated area visible for apple, pear, and eggplant which grew and intensified over time. Within these rapidly developing samples, apple showed the best results regarding a well-defined electroporated area. Potato showed a similar pattern, but it took longer for the brown electroporated area to become visible. Compared to the control sample, the electroporated area within the carrot became softer with a wet surface. The sweet potato did not show clear visual signs of EP. Kohlrabi, zucchini, cucumber, and celeriac did show subtle discoloration of the electroporated area over time. However, the electroporated area in the already light-coloured samples did not darken as expected and instead turned into a whitish colour, resulting in a lack of contrast 6 h post EP, all fruits and vegetables started to show signs of shrinkage due to drying after the cut.

**Figure 3.**
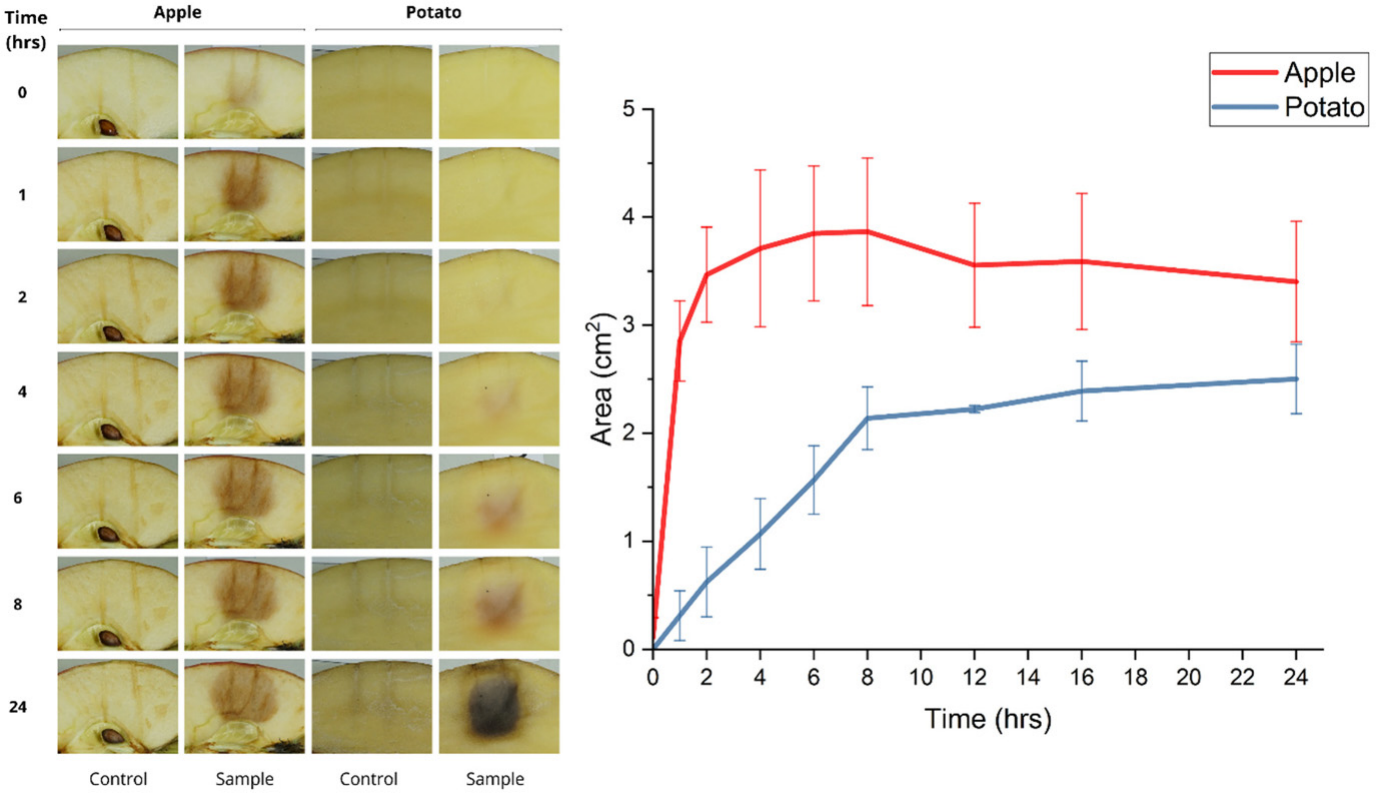
Left: Visual representation of apple and potato at 0, 1, 2, 4, 6, 8, and 24 h post EP including controls with needle impression. Right: Electroporated area in cm^2^ of the apple (red) and potato (blue) 0, 1, 2, 4, 6, 8, 12, 16, and 24 post EP. EP pulse parameters included 1000 V, 70 pulses, 100 *µ*s pulse length, and 100 ms interval with a 1 cm distance between the two-needle electrodes. Values and error bars represent (*N* = 5) and SD.

Out of all samples, the apple and potato visually showed the most well-defined brown electroporated area. Therefore, their electroporated area in cm^2^ was determined in *N* = 5 samples each at time points of 0, 1, 2, 4, 6, 8, 12, 16, and 24 h post-EP (Figure 3). For every time point, apple showed a larger electroporated area than potato. Directly after EP (0 h), the average electroporated area of apple was 0.11 ± 0.18 cm^2^, compared with 0 ± 0 cm^2^ for the potato. The electroporated area in apple increased over time and started to stabilize 2 h post-EP (3.47 ± 0.44 cm^2^). The area reached a maximum of 3.87 ± 0.68 cm^2^ at 8 h post-EP. The electroporated area in potato increased more gradually over time than apple, and started to stabilize 8 h post-EP (2.14 ± 0.29 cm^2^). The area reached a maximum of 2.50 ± 0.32 cm^2^ at 24 h post-EP.

### Comparison between plant-based model and in-vivo animal data

**Figure 4.**
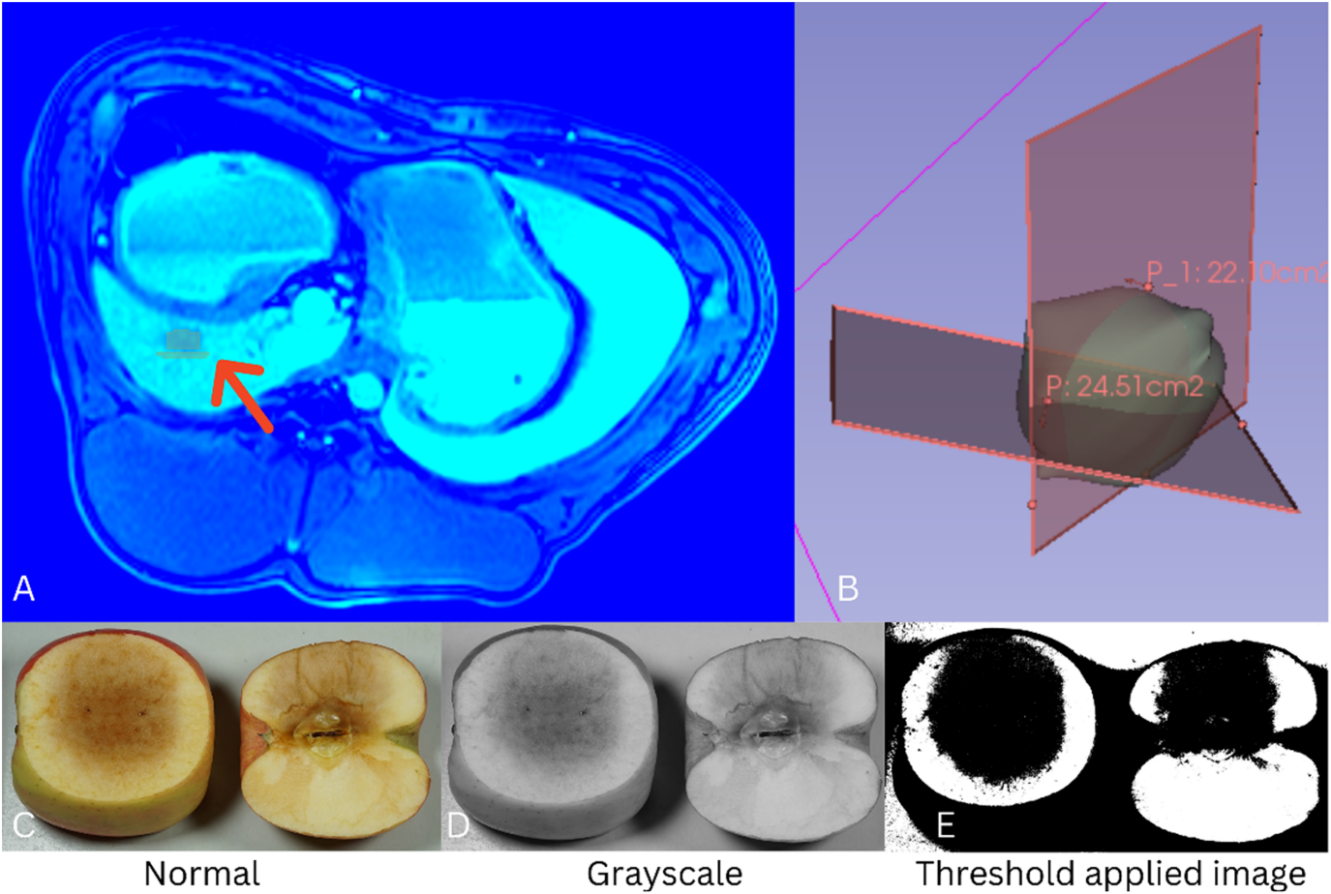
A: segmented MRI of electroporated swine liver using slicer; B: measurement of the electroporated area in swine liver; C: A representational non-processed image of apple 2 h after EP sliced in orthogonal (*N* = 5) and parallel (*N* = 5) plane of the needle; D: grayscale mode conversion using GIMP 2.10; E: threshold applied image of apple for calculating the electroporated area by counting pixels. EP pulse parameters included 3000 V, 70 pulses, 100 *µ*s pulse length, and 100 ms interval with a 2 cm distance between the two-needle electrodes where the exposed needle length was 1.5 cm.

After exposure to an electric potential of 3000 V, 70 pulses, 100 *µ*s pulse length, and 100 ms interval, the electroporated area in the swine liver and apple was determined (Figure 4).

Qualitatively the electroporated area of apple and swine liver both showed a spherical geometry. Within the swine liver, the electroporated area was found to be 24.51 cm^2^ in the parallel plane to both electrodes, and 22.10 cm^2^ in the orthogonal plane to both electrodes. For the apple, the electroporated area was 8.30 ± 0.13 cm^2^ in the parallel plane, and 14.80 ± 2.12 cm^2^ in the orthogonal plane to the electrodes.

## Discussion

A wide variety of fruits and vegetables were selected for the experiment based on their size, geometry, and consistency. For the vegetables, we covered different parts of plant modification, like root modification (sweet potato, carrot, and celeriac) and stem modification (potato and kohlrabi). The shelf life of potatoes will affect their water content, therefore freshly bought potatoes were included. Cucumber, zucchini, eggplant, apple, and pear were selected based on their sturdiness and uniformity of flesh inside for better visibility of the electroporated area after EP. For the animal data, swine liver was chosen because it is a well-accepted model for human liver tissue.^24^

For the initial screening of the plant-based samples, a two-point needle electrode design was chosen to compare the electroporated area. For the animal experiment, the NanoKnife system only approved the use of two needles concurrently, so a similar setup was also used to electroporate the apples to compare the geometry and size of the electroporated areas.

The pulse parameters of the initial screening of the fruits and vegetables, as well as the comparison of apple with swine liver, were chosen because they are in the range of clinical IRE treatment of cancer.^25^ Some types of fruit and vegetables did not show any visual changes after EP. The efficiency of pulsed electric field treatment depends on chemical composition, electrical properties of biological tissues, and the induced electric field.^16^

To make sure that the browning of the electroporated area is not a result of heating but can be attributed to EP-induced cell death, the temperature around the location of the electrode tips was probed directly after EP with a thermocouple. The maximum temperature (34°C) was not enough to induce cell death due to the heating effect, so no additional studies on the temperature were carried out.

Mechanical damage due to the insertion of the needles can also contribute to discoloration around the needles. Due to similar needle parameters, e.g. sharpness, the mechanical damage caused by all needles should, however, be the same. Therefore, the visually observed browning of the fruit and vegetable samples mainly indicates the electroporated area.

**Figure 5.**
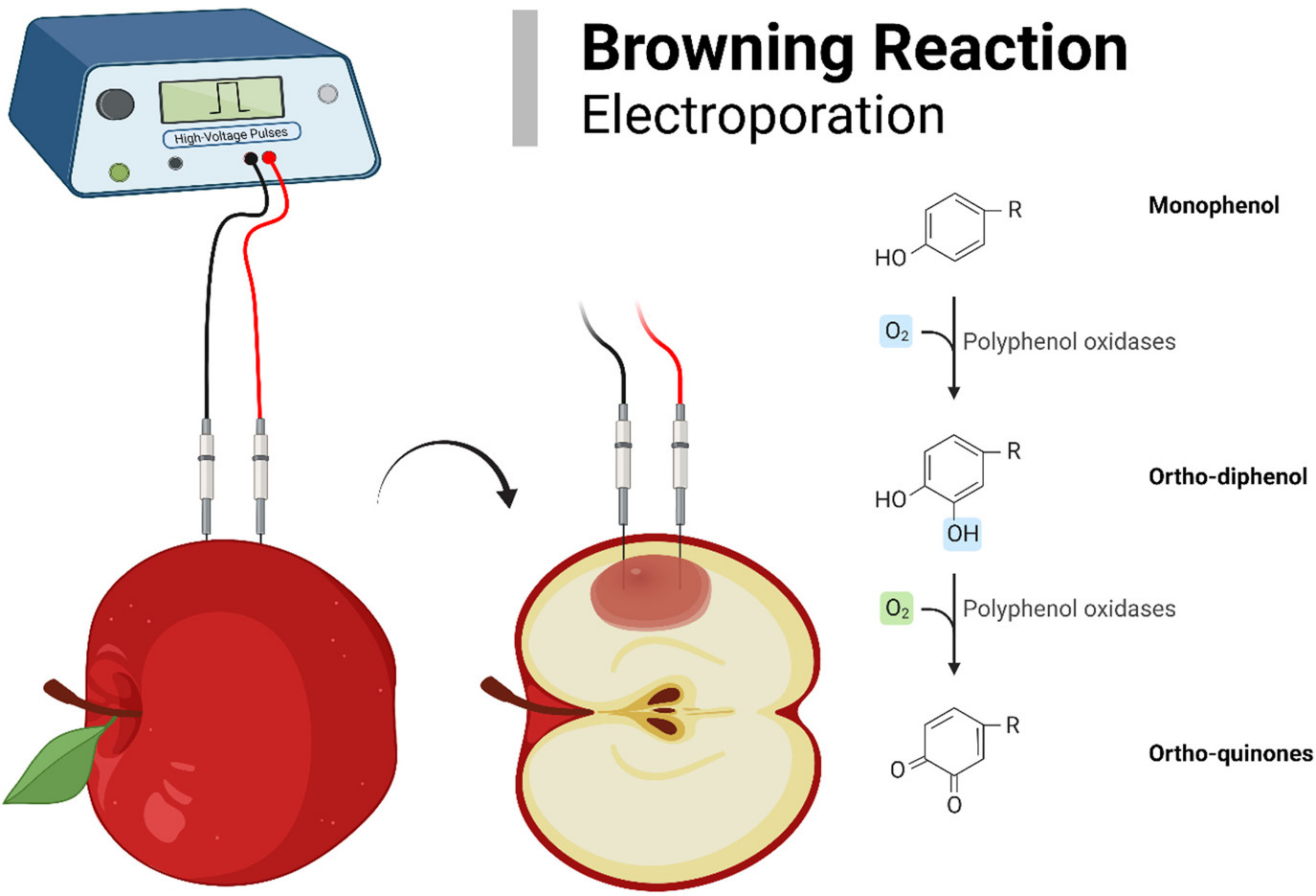
Browning reaction in apple after EP.

During irreversible EP, the membrane is disrupted, making polyphenol oxidase, a group of Cu-containing enzymes, available for oxidation. Polyphenol oxidases oxidize several phenols in the presence of oxygen molecules.^26^ The final product of this oxidation process is o-quinones (Figure 5). O-quinones are highly reactive transition state complexes and undergo non-enzymatic reactions. This makes the brown complex polymers generally known as melanins, which cause the browning effect in fruits and vegetables.^27, 28^

Molecular oxygen, a necessary reagent for browning, is present inside the fruits and vegetables to maintain the metabolism. Even after the harvesting, fruits, and vegetables will intake oxygen for respiration to generate energy (ATP) and cell viability.^28, 29^ In the case of apples, by structure, there are void spaces in the tissue with gas-filled intercellular spaces. Tomographic images obtained by Synchrotron X-Ray Computed Tomography (CT) verified the presence of gas-filled intercellular spaces. The air sacs in apple were found to be as small as 1 *µ*m, with a void fraction in the apple cortex of 23%.^30^

The concentration of chloroplast contacting enzyme, polyphenol oxidase, and molecular oxygen vary between different fruits and vegetables. From the literature survey, polyphenol oxidase is verified in potato, apple, pear, and eggplant.^26–28^ This concentration difference determines the rate of browning in fruits and vegetables. This might explain why in particular, these samples visually showed a brown electroporated area immediately after EP within our study.

Over the past years, potatoes have been used as a plant-based model for studying the effects of IRE.^17–19^ In this study, for potatoes, it took at least 8 h post-EP to visually show a well-defined brown electroporated area without any advanced visualisation methods. This data is in line with literature.^20^ The present study suggests that apple could be an alternative plant-based model instead of potatoes for IRE studies. The significant benefits are that it takes less time, within 2 h, to visually define the brown electroporated area after EP without any advanced visualisation methods (Figures 2 and 3). Apples can also be used with higher electrical field strengths than potatoes. The potato could not be used for the comparison with in-vivo data, as it showed sparks while applying 3000 V. This makes apples a better model for EP device development and testing.

While splitting open the plant-based samples just after the EP, especially carrot, an unusual increase in water content was observed at the surface. After 6 h post EP, the samples showed signs of shrinkage, almost complete shrinkage was observed at the 24-h timepoint. When the water content in these fresh vegetables (no matter whether it is electroporated or not) exposes to the atmospheric air, it evaporates. This evaporation rate also depends on the atmospheric vapour pressure, the temperature of the evaporating surface, evaporation surface area, etc ^31^ The electroporated tissue will have extra water leakage from the intercellular matrix to outer cellular space due to the increase in permeability of the membrane after EP. This process is facilitated by the normal diffusion mechanism and causes more shrinkage within the electroporated area, as seen in Figure 2.

For the comparison of plant-based tissue and in-vivo animal data, the electroporated area in the swine liver, 24.51 cm^2^ parallel plane, and 22.10 cm^2^ orthogonal plane, and apple, 8.30 ± 0.13 cm^2^ and 14.80 ± 2.12 cm^2^ respectively, were compared and 2.12. Within the apple, the smaller size of the parallel electroporated area can be explained by the exclusion of the seed-containing-core. Although the sizes of the electroporated areas between swine liver and apple differ, both values are still in a comparable range. It should be stressed that only one swine datapoint could be used for this comparison. Electric field distribution in living tissue, both plant-based and animal, depends upon electrical parameters like conductivity and relative permittivity, mainly driven by ionic concentration and water content.^32^ From electrical impedance spectroscopy measurements, it was found that these electrical parameters will fluctuate from one biological sample to another.^16^ For the same sample, these values will also change drastically in time, adding up to the complexity of quantitatively predicting the electroporated area. Qualitatively the electroporated area of apple and swine liver both showed a spherical geometry, which can be explained by the distribution of the field lines during EP.^21, 33^

Other than the minor limitations already mentioned, another issue arose during the analysis of the images. Since the peaks of the histogram for potatoes will vary from sample to sample, it was challenging to develop a generalized automated method for threshold selection to segment the electroporated area in the screening of a suitable plant-based model. Due to the fact that sample colours varied within the species as well as over time, selecting a single threshold value for all samples were impossible. Therefore, we opted for a subjective, manual, visual-based selection of the EP zone for the screening of the plant-based samples. However, in the case of apples, a static threshold, histogram-based, generalised segmentation was possible. Therefore, in apples, this semi-automatic analysis method was implemented in comparison to in-vivo data.

To conclude, potato and apple are suitable plant-based models for the visual evaluation of electroporated areas after irreversible EP, with apple being the best choice for fast visual results and automated analysis. Qualitatively, the electroporated area of apple and swine liver both showed a spherical geometry. Based on the initial comparison shown in this article, the size of the electroporated area of the apple may be promising as a quantitative predictor in animal tissue. Future research should investigate this point in more detail, for example including different electrode configurations and pulse parameters. Even if plant-based models cannot completely replace animal experiments, they can be used in the early stages of EP device development and testing, decreasing animal experiments to the necessary minimum.
